# Novel Animal Defenses against Predation: A Snail Egg Neurotoxin Combining Lectin and Pore-Forming Chains That Resembles Plant Defense and Bacteria Attack Toxins

**DOI:** 10.1371/journal.pone.0063782

**Published:** 2013-05-30

**Authors:** Marcos Sebastián Dreon, María Victoria Frassa, Marcelo Ceolín, Santiago Ituarte, Jian-Wen Qiu, Jin Sun, Patricia E. Fernández, Horacio Heras

**Affiliations:** 1 Instituto de Investigaciones Bioquímicas de La Plata (INIBIOLP), Universidad Nacional de La Plata (UNLP) – Consejo Nacional de Investigaciones Científicas y Técnicas (CONICET CCT-La Plata), La Plata, Argentina; 2 Instituto de Investigaciones Físico-Químicas, Teóricas y Aplicadas (INIFTA), UNLP - CONICET CCT-La Plata, La Plata, Argentina; 3 Cátedra de Bioquímica y Biología Molecular, Facultad de Ciencias. Médicas, UNLP, La Plata, Argentina; 4 Department of Biology, Hong Kong Baptist University, Hong Kong, P. R. China; 5 Instituto de Patología B. Epstein, Cátedra de Patología General Veterinaria, Facultad Cs. Veterinarias, UNLP, La Plata, Argentina; 6 Facultad de Ciencias Naturales y Museo, UNLP, La Plata, Argentina; Ecole Polytechnique Federale de Lausanne, Switzerland

## Abstract

Although most eggs are intensely predated, the aerial egg clutches from the aquatic snail *Pomacea canaliculata* have only one reported predator due to unparalleled biochemical defenses. These include two storage-proteins: ovorubin that provides a conspicuous (presumably warning) coloration and has antinutritive and antidigestive properties, and PcPV2 a neurotoxin with lethal effect on rodents. We sequenced PcPV2 and studied whether it was able to withstand the gastrointestinal environment and reach circulation of a potential predator. Capacity to resist digestion was assayed using small-angle X-ray scattering (SAXS), fluorescence spectroscopy and simulated gastrointestinal proteolysis. PcPV2 oligomer is antinutritive, withstanding proteinase digestion and displaying structural stability between pH 4.0–10.0. cDNA sequencing and protein domain search showed that its two subunits share homology with membrane attack complex/perforin (MACPF)-like toxins and tachylectin-like lectins, a previously unknown structure that resembles plant Type-2 ribosome-inactivating proteins and bacterial *botulinum* toxins. The protomer has therefore a novel AB toxin combination of a MACPF-like chain linked by disulfide bonds to a lectin-like chain, indicating a delivery system for the former. This was further supported by observing PcPV2 binding to glycocalix of enterocytes *in vivo* and in culture, and by its hemaggutinating, but not hemolytic activity, which suggested an interaction with surface oligosaccharides. PcPV2 is able to get into predator’s body as evidenced in rats and mice by the presence of circulating antibodies in response to sublethal oral doses. To our knowledge, a lectin-pore-forming toxin has not been reported before, providing the first evidence of a neurotoxic lectin in animals, and a novel function for ancient and widely distributed proteins. The acquisition of this unique neurotoxic/antinutritive/storage protein may confer the eggs a survival advantage, opening new perspectives in the study of the evolution of animal defensive strategies.

## Introduction

Escaping predation is essential to survival. To reduce predation, organisms have developed an array of chemical and physical defensive strategies, but predators in turn have evolved adaptive mechanisms to overcome these defenses in a battle of coevolving prey defenses and predator counter-defenses [1]. Eggs are perhaps the most endangered stage in the life cycle of an animal. Motionless and often conspicuous, eggs are highly vulnerable to both predators and parasites [2] and their high nutritional value makes them subject to intense predation. Consequently, many invertebrates defend their eggs by endowing them with deterrent chemicals as has been well documented in insects that sequester toxic compounds from plants [3]–[5] and in some terrestrial and marine gastropods [6], [7]. There are, however, a few eggs that are winning in the “arms-race” and escape intense predation such as the aerial egg clutches from the aquatic apple snail *Pomacea canaliculata* (Lamarck, 1822) (Caenogastropoda, Ampullariidae) which, while filled with large amounts of carbohydrates and storage proteins (perivitellins) [8], have only one reported predator worldwide, the fire ant *Solenopsis geminata* (Fabricius, 1804) [9]. Females from this freshwater apple snail deposit clutches of hundreds of pink-reddish eggs that are unusual in two respects: they are cemented outside the water and they are brightly colored [10], [11]. The conspicuous egg coloration presumably advertises to visual-hunting predators the presence of egg defenses (aposematic or warning coloration), a strategy used by noxious organisms to visually communicate their toxicity or distastefulness to potential predators [12]. Biochemical evidence indicates that apple snail perivitellins are involved in egg defense against predation [13]. These proteins, synthesized in the female albumen gland, are deposited around the fertilized ovocyte as part of the perivitelline fluid [14]. Two of these perivitellins, PcPV2 (previously called PV2) and ovorubin (PcOvo), are neurotoxic and diminish rat growth rates, respectively [8], [13], [15]–[17]. PcPV2 is a neurotoxin with a strong lethal effect on the central nervous system of mice that induces changes in calcium homeostasis and results in the apoptosis of selected neuron populations. Structural studies have shown that PcPV2 is a globular, compact and well-folded glyco-lipoprotein, with 2.5% w/w carbohydrates [18]. This 400 kDa oligomer is an octamer of four 98 kDa heterodimers, each composed of a 67 kDa heavy chain (PcPV2-67) and a 31 kDa light chain (PcPV2-31). The heavy and light chains are held together by disulfide bonds and the heterodimers are assembled into native PcPV2 by non-covalent forces [18]. PcPV2 is the only reported genetically encoded toxin located inside an egg [17].

PcPV2 is synthesized and secreted together with the other identified component of the apple snail egg defenses, the carotenoprotein PcOvo. This protein is a multifunctional perivitellin that is massively accumulated in the perivitelline fluid and is involved in protection against abiotic stressors [19], as well as against predators. Besides providing conspicuous coloration to eggs, PcOvo defends the embryos against predators by reducing the digestibility and nutritional quality of the eggs [13], [20]. The recently described proteome of *P. canaliculata* perivitellin fluid revealed more potential defensive proteins [21].

Unlike this comprehensive body of information on PcOvo, information is lacking about the capacity of the neurotoxin PcPV2 to withstand the harsh gastrointestinal environment or about its ability to reach a predator’s circulatory system, both of which are critical steps allowing the toxin to reach the central nervous system. This, together with the lack of sequence data for egg neurotoxins, motivated the present study. Through a combination of biochemical, biophysical, histopathological, molecular biology, and cell biology experiments, we show that PcPV2 consists of a membrane attack complex/perforin-like heavy chain covalently linked to a lectin-like light chain. We provide evidence showing that its native structure is rather stable in the pH range of the gastrointestinal tract and that it is able to withstand simulated gastrointestinal digestion. The toxin is a functional lectin and shows no hemolytic activity, interacts with rat small intestine epithelia *in vivo* and with intestinal cells in cultures, and would be able to reach the circulation in rats. These results provide the first clues about the mechanisms that allow the neurotoxin to enter a predator’s body. To our knowledge, the presence of a neurotoxic lectin with a dichain structure able to traverse the intestinal barrier has not been reported in animals.

## Methods

### Ethics Statement

All the studies performed with rats, mice and rabbits were approved by the Directive Board of the INIBIOLP and were carried out in accordance with the Guide for the Care and Use of Laboratory Animals [22]; (Instituto de Investigaciones Bioquímicas de La Plata’s Animal Welfare Assurance No. A5647–01).

### PcPV2 Isolation and Purification

Egg masses of *P. canaliculata* with embryos developed to no more than the morula stage were employed. Eggs were homogenized with 20 mM Tris/HCl, pH 6.8 buffer, containing a protease inhibitor cocktail (Sigma Chemicals, St. Louis). The crude homogenate was centrifuged sequentially at 10,000×g for 30 min and at 100,000×g for 50 min. PcPV2 was isolated from the supernatant by ultracentrifugation and purified by HPLC using a Mono Q HR 10/10 column as detailed previously [23]. Proteins were quantified [24] and purity was checked by PAGE.

### Cloning of PcPV2 Subunits

Total RNA was extracted from albumen gland (AG) using Trizol (Invitrogen, Carlsbad, USA) according to the manufacturer’s procedure. The first strand cDNA synthesis from total RNA (2 µg) was performed using oligo dT and M-MLV reverse transcriptase (USB, Cleveland, USA). N-terminal sequences of both subunits (NCBI accession No.: P0C8G7 for PcPV2-67 and P0C8G6 for PcPV2-31 [14]) were used to search against the *P. canaliculata* transcriptome using tBLASTn [25]. The matched unigenes were used to design primers for 3′RACE (Rapid Amplification of cDNA Ends) to obtain the full open reading frames (ORF). The PCR products were checked by agarose gel electrophoresis, purified and ligated into the pMD-18T plasmid (Takara, Dalian, China). This plasmid was sequentially transformed in *E.coli* competent cells for subsequent DNA sequencing.

Sequences were deposited in GenBank (accession No: JX155861 for PcPV2-67 and JX155862 for PcPV2-31.

### Sequence Analysis

Signal peptides of each protein were predicted by the SignalP 4.0 Server [26]. Kinase specific eukaryotic protein phosphorylation sites were predicted by the NetPhosK 1.0 Server under a filter threshold of 0.85. N-Glycosylation sites were predicted with Net-Glyc 1.0.

The translated amino acid sequences were used for phylogenetic analysis using MrBayes (v.3.2.0) with a mixed amino acid model [27]. Bayesian analysis was performed with four chains of 100,000 generations. The tree was sampled every 100 generations, and the final burnin value was set to 20,000. The standard deviation of the split frequencies fell below 0.01. The tree was visualized by Treeview (v.1.6.6).

### PcPV2 Stability with Regard to pH

PcPV2 (0.24 mg/mL) at different pH values (2.0 to 12.0) were prepared using sodium phosphate salts and citric acid buffers [28]. After 48 h of incubation, samples were analyzed by light scattering and fluorescence spectroscopy (see below).

### Small Angle X-ray Scattering (SAXS)

SAXS experiments were performed at the D02A-SAXS2 line operating in the Laboratório Nacional Luz Sincrotron, Campinas (SP, Brazil). The scattering pattern was detected using a 2D-MARCCD charge coupled device assisted by FIT 2D software {Hammersley, 1997 12369/id}. The wavelength of the beam was kept to 1.448 Å, and the sample-to-detector distance was 1044 mm, allowing a nominal Q-range between 0.012–0.25 Å^−1^. The temperature was stabilized at 15°C using a circulating water bath.

Corrections for beam intensity, detector homogeneity and sample absorption were performed following standard procedures. At least three independent curves were averaged for each single experiment. The size of PcPV2 was determined using the gyration radii (*R*_G_) obtained by analysis of SAXS patterns as Guinier plots (ln(I) = ln(I_0_)–*R*_G_Q^2^/3, Q = 4πsin(θ)/λ, *R*_G_Q≤1) and the globularity evaluated by inspecting the Kratky plot (I(Q)*Q^2^ versus Q). The Pair Distance Distribution Function (PDDF) was evaluated using the regularized Fourier transformation method implemented in the program GNOM 4.5 [30].

### Fluorescence Spectroscopy

Tryptophan fluorescence spectra of PcPV2 (1.2 µM) at each pH (2.0 to 12.0) were recorded in emission scanning mode in an Olis-upgraded SLM 4800 (Bogart, GA). Tryptophan emission was excited at 290 nm (4-nm slit) and recorded between 310 and 410 nm (4-nm slit). Fluorescence measurements were performed in 5-mm optical path length quartz cells at 20°C. Each spectrum was corrected for buffer fluorescence and averaged from at least two independent runs.

### Gel Electrophoresis

Denaturing electrophoresis was performed on a 4–20% gradient SDS-PAGE using β-mercaptoethanol as reducing agent and stained with Coomassie Brilliant Blue R-250 (Sigma Chemical Co, USA).

### PcPV2 Isoelectric Point Determination by 2-dimensional Electrophoresis

Two-dimensional electrophoresis (2-DE) isoelectric Focusing (IEF) was performed using an Ettan IPGphor III (GE Healthcare, Uppsala, Sweden) and 7-cm linear pH 4–7 immobiline dry strips (GE Healthcare). Strip rehydration and loading were carried out overnight at room temperature in a dilution buffer containing 1 µg of PcPV2. PAGE electrophoresis was carried out at 15 mA/gel and spots visualized by Coomassie Brilliant Blue R-250.

For the 2-DE SDS-PAGE, immobiline strips were immersed in 7 M urea, 2 M thiourea, 2% CHAPS, 0.5% v/v IPG buffer 4–7 linear containing 3 µg protein. After IEF, the immobiline dry strips were equilibrated at room temperature for 20 min in a buffer containing: 75 mM Tris–HCl, 6 M urea, 30% v/v glycerol, 2% w/v SDS, 0.002% w/v bromophenol blue and 1% w/v DTT, and then alkylated for 20 min in the above buffer, but with 4.5% w/v iodacetamide in place of DTT. The second-dimension was carried out in 12% polyacrylamide gels.

### In vitro PcPV2 Digestibility

The simulated gastrointestinal digestion of PcPV2 was performed following the method described by Moreno [31] with slight modifications. Briefly, gastric digestion was performed at 37°C for 120 min at pH 2.5 in the presence of porcine pepsin (Sigma, UK) at a ratio of enzyme: substrate 1∶20 (w/w). Aliquots were taken at 0, 60 and 120 min and analyzed by SDS-PAGE using β-mercaptoethanol as reducing agent. The digestion was stopped by raising the pH to 7.5 using 50 mM phosphate buffer. For *in vitro* duodenal digestion the 120 min gastric digest was used as starting material using trypsin from bovine pancreas (Sigma) at a ratio of enzyme: substrate 1∶400 (w/w), at 37°C taking aliquots at 0, 60 and 120 min for SDS-PAGE analysis as described above. Albumin was used as positive (with enzyme) and negative (without enzyme) controls in both gastric and duodenal digestions.

### Binding Assay to Rat Intestinal Tissue

PcPV2 binding to epithelial cells of the small intestine *(in vivo* test) was performed using male Wistar rats from the Animal Facility Colony of the Faculty of Medical Sciences, UNLP. Rats came from a colony started with the strain WKAHlHok (Hokkaido University, Japan). Animals weighing 150±2 g were housed in cages with a 12∶12 l:d cycle at 22±1°C and 45–60% relative humidity. Rats (n = 6) were orally administered 100 µl of egg extract (8 mg of total protein) in 50 mM phosphate buffer (pH 7.4) on a daily basis, and a control group was administered the same amount of buffer without the toxin. After 4 days, they were euthanized by CO_2_ inhalation in a closed chamber. CO_2_ was slowly piped into the chamber, so that the animals were exposed to a gradually rising gas concentration to ameliorate suffering. The first part of small intestine was cut, washed 6 times with PBS to remove food and non-bound protein, and fixed in 4% phosphate buffered formalin (pH 7.0) for histological examination. Cylindrical tissue samples of the small intestine were post fixed in formaldehyde and embedded in paraffin wax. Five tissue sections from each animal were assayed by immunohistochemistry (IHC). Tissue sections from control and treated animals were incubated with rabbit polyclonal anti-PcPV2 serum (1∶100 dilution) previously prepared [14], and revealed with the Envision plus kit (Dako, Carpinteria, CA, USA). Positively immunostained regions showed a golden dark brown color using 3,3′-diaminobenzidine tetrahydrochloride (DAB) and H_2_O_2_ reaction substrates. All sections were counterstained with Maeyer hematoxylin.

### Binding Assay to Intestinal Epithelial Cells in Culture

PcPV2 was labeled with the Alexa Fluor 488 Protein Labeling Kit (Life Technologies-Molecular Probes) according to manufacturer’s instructions. Labeled bovine serum albumin (BSA) was employed as a negative control.

Human colorectal adenocarcinoma cells (Caco-2) obtained from the ATCC (Cedarlane Inc., Burlington, ON) were cultured in Dulbecco’s modified Eagle’s medium (DMEM) (4.5 g/liter D-glucose) supplemented with 10% newborn calf serum, penicillin/streptomycin, amino acids and vitamins (Life Technologies-Invitrogen). Cells were cultured at 37°C in a humidified atmosphere of 5% CO_2._ Culture medium was replaced every 2 days and subcultured by trypsination when 95% confluent. Cell viability of every preparation exceeded 90% as determined by trypan blue exclusion, counting the stained cells.

For the PcPV2 treatment, harvested cells were seeded on 24-well plates (Orange Scientific, Belgium), at approximately 80% confluence in controls at the end of a 2-day experiment. The culture medium was replaced daily and after 48 h incubation, cells were washed twice with PBS and incubated with an Alexa488-labeled PcPV2 preparation in PBS (400 µg/ml) for 1 h at 37°C. Cells were observed in an inverted fluorescence microscope (Olympus IX-71). Negative control wells were incubated with an Alexa488-labeled BSA in PBS (400 µg/ml). All protein preparations used in cell culture assays were sterilized by filtration (0.22 µm).

### Hemagglutinating and Hemolytic Activity

Rabbit erythrocytes were obtained from the animal facilities at University of La Plata (UNLP). Blood samples were obtained by venous puncture and collected in sterile Elsever's solution (100 mM glucose, 20 mM NaCl, and 30 mM sodium citrate, pH 7.2) (Sigma-Aldrich, St. Louis, MO, USA). Prior to use, erythrocytes were washed by centrifugation at 1500×g for 10 min in 20 mM phosphate buffer, 150 mM NaCl, pH = 7.4. This procedure was repeated several times until the supernatant remained clear. Hemagglutinating activity was assayed in microtiter U plates (Greiner Bio One, Germany) by incubating a two-fold serial dilution of PcPV2 (1.6 g/L) with 1% erythrocyte suspension in phosphate buffer at 37°C for 2 h. Results were expressed as the inverse of the last dilution showing visible hemagglutinating activity at naked eye [32]. Hemolytic activity was followed measuring the release of hemoglobin spectrophotometrically at 412 nm in the supernatant of the wells [33].

### Rat and Mice Oral Immunization Analysis

To check if the toxin was able to reach predator’s circulatory system, rats (n = 5) were subjected to an oral, sublethal administration of egg extract containing 3 mg protein in PBS (approx. 400 µg PcPV2), with a second dose of 3 mg after 19 days. Adjuvants were not used. Serum was harvested 4 days later and kept at −70°C until used. Spots of 1 µg PcPV2 toxin were pipetted onto nitrocellulose membranes (Amersham). After blocking for 1 h at 37°C with 5% (w/v) non-fat dry milk in PBS–Tween, the membranes were incubated overnight at 4°C with anti-sera dilutions in 1% (w/v) non-fat dry milk in PBS–Tween from control and PcPV2 treated animals. The specificity of rat serum towards PcPV2 was detected using an anti-rat IgG horseradish peroxidase conjugate (Bio Rad Laboratories, Inc.). Immunoreactivity was visualized by electrochemiluminescence.

In another study, 20±2 g BALBc female mice (n = 12) were given an oral, sublethal dose containing 3 mg of egg extract in PBS, with a second dose of 3 mg protein in PBS after 7 days. No adjuvants were employed. After 14 days, control (n = 6,) and treated (n = 6) mice were injected i.p. with 40 µg of purified PcPV2, a dose well above its reported LD50, 96 h of 0.25 mg/kg, i.p. (5 µg/mice) [17]. Control and treated mice were observed every 6–8 h for 96 h and behavioural changes monitored. As soon as flaccid paralysis of the outstretched rear limbs appeared or their condition severely deteriorated, animals were recorded as “dead” and euthanized by CO_2_ inhalation as described above.

### Statistical Analysis

Data were analyzed by one way analysis of variance (ANOVA). When p values were <0.05, the significance between groups was estimated by Tukey’s test.

## Results

### PcPV2 Primary Structure Features

Cloning of the full-length cDNA of the two PcPV2 subunits allowed the primary structure analysis. When compared with sequences available in GenBank, the PcPV2-31 and PcPV2-67 sequences displayed the highest identity with tachylectin-1 from the lancelet *Branchiostoma belcheri* (PcPV2-31) and a MACPF from the Mediterranean mussel *Mytilus galloprovincialis* (PcPV2-67) (E-values of 1 e−20 and 3 e−88, respectively).

**Figure 1:**
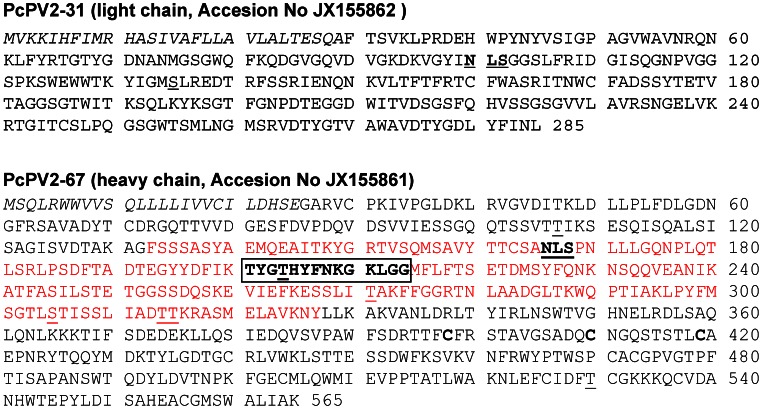
Deduced aminoacid sequences of light (A) and heavy (B) PcPV2 subunits. The putative signal sequences are in italics, MACPF domain is marked in red and MACPF signature is boxed. Potential phosphorylation sites are underlined, and potential glycosylation sites are underlined and in bold.

The translated PcPV2-31 protein consists of 285 residues with a putative signal sequence of 29 residues. The cleavage site, predicted to be at position 29–30, is coincident with N-terminal sequencing of the protein by Edman degradation [14], which confirms that the first residue of the small/light subunit is Phe (Figure 1A). After removing the signal peptide, the mature protein has a calculated molecular mass of 28.2 kDa and an estimated isoelectric point (pI) of 8.69, while 2-dimensional electrophoresis (2-DE) analysis showed the presence of several isoforms of different pI ranging from ∼4.7–9, [Figure S1]. The protein displays a single N-link glycosylation site (NXS/T) predicted at [N-99] and a protein kinase C phosphorylation site predicted at [S-135]. The nucleotide sequence reported here was deposited in the GenBank data bank with accession No JX155862.

The PcPV2-67 subunit contained 565 translated residues and the first 25 amino acid residues encoded a putative leader sequence, with a predicted cleavage site between position 25 and 26 (Figure 1B). This result is consistent with the previously reported PcPV2-67 N-terminal sequence [14] confirming that the first amino acid of the mature protein is Ala. After removing the signal peptide, the theoretical molecular mass of the mature subunit was 59.5 kDa and the pI was 5.31, similar to the experimental pIs determined by 2-DE (pI ∼5.2, Figure S1). The nucleotide sequence was deposited in the GenBank data bank with accession No. JX155861.

The heavy chain has one predicted N- glycosylation site (NXS/T) at [N-166] that according to Net-Glyc 1.0 has a low probablity of being glycosylated. Regarding phosphorylation, seven potential protein kinase C phosphorylation sites at [T-107], [T-204], [T-271], [S-305], [T-314], [T-315], and [T-530] were predicted (Figure 1B).

Pfam software analysis indicated that PcPV2-67 is a member of the MACPF superfamily cl02616. (**pfam** score 1e-21). Analysis also showed the presence of a MACPF domain between residues 131 and 327. The apple snail protein showed the conserved signature motif, Y/W-G-T/S-H-F/Y-X6-GG, included in all members of the MACPF family (Figure 1B and Figure S2). This motif is more conserved than that of *M. galloprovincialis* MACPF, where Y/W is mutated to F (Figure S2). All cysteine residues and two GG sites (213–214, 276–277) that are assumed to be important for MACPFs membrane binding abilities are also conserved in PcPV2-67 which also shares with *M. galloprovincialis,* C-terminal region rich in C. Like *M. galloprovincialis*, the *P. canaliculata* MACPF does not have a calcium-dependent membrane-binding (C2) domain, although it has one of the important residues of that domain (T395) [34] ().

### Phylogenetic Analysis

**Figure 2:**
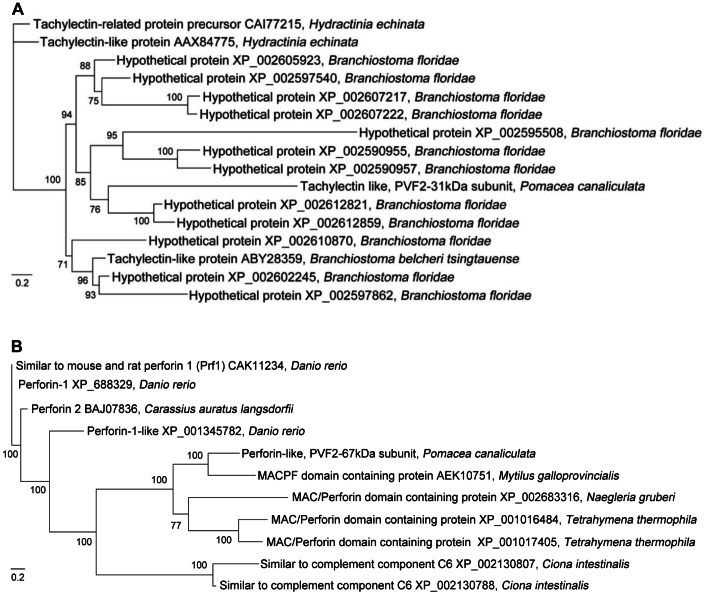
Bayesian phylogenetic tree of light (A) and heavy (B) PcPV2 subunits of *Pomacea canaliculata*. Numbers above branches represent Bayesian posterior probabilities of finding a given clade. Scale bar, 0.2 nucleotide substitution.

BLASTx analysis using the NCBI database revealed that the heavy and light subunits matched to 10 sequences from 6 species and 15 sequences from 3 species (1e-5), respectively. These sequences were further subjected to phylogenetic analysis (Figure 2). The sequences that resemble the PcPV2-67 subunit came from highly divergent taxa ranging from the protist, *Tetrahymena thermophila* to the zebrafish *Danio rerio*, indicating independent origins of these MACPF proteins. The sequences that resemble the PcPV2-31 subunit fall into a clade with several lancelet sequences, which may indicate an early duplication of the tachylectin-like chain during the divergence of chordates.

### Structural Stability Against pH

**Figure 3:**
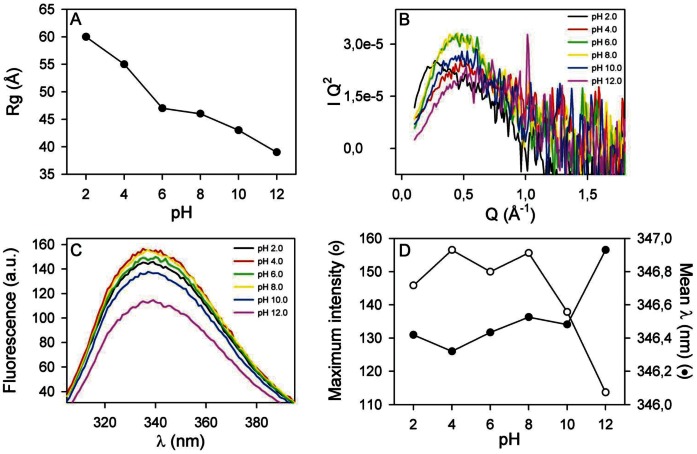
Effect of pH on PcPV2 structure as determined by SAXS (A, B) and Trp fluorescence (C, D). A: PcPV2 radius of giration Rg as a function of pH. B: Kratky plot of data depicted on Figure 2A that highlights shape variations of PcPV2. C: Trp fluorescence emission spectra at different pH values. D: Mean wavelength of the spectrum (closed circles) and maximum intensity (open circles). Mean wavelength refers to Σ(λ F(λ))/Σ F(λ) where F(λ) corresponds to the fluorescence intensity for a given λ. Error bars in A fall within the size of the points; error bars in B and C never exceeded 1% of the value; error bars in D are 2 nm for wavelengths and 10% for signal intensity.

As gastrointestinal pH extremes are one of the first stresses that PcPV2 must face when ingested, we studied its behavior in a wide pH range. The native PcPV2 gyration radius**,** Rg, at different pH values, determined by small angle X-ray scattering (SAXS), is shown in Figure 3A where a slight decrease at pH >8.0 can be observed. Below the experimentally determined pI value (∼6.2) a steady increase in Rg is observed possibly due to unspecific aggregation of PcPV2. A similar tendency can be seen in the pH evolution of the PDDF [Figure S3]. The steady decrease in Rg under alkaline pH conditions can be attributed to subunit disassembly. The Kratky plots also did not show a dramatic loss of globularity even at both pH extremes (Figure 3B).

The tryptophan fluorescence spectra between pH values of 2.0 and 12.0 (Figure 3C, D) did not show a shift of its emission maxima except for a slight red shift at pH 12.0. An intensity decrease was evident at pH >8.0, indicative of the exposure of some of the tryptophan residues to the aqueous environment. Together with the pH behaviour of Rg, we could conclude that the structure of PcPV2 is rather stable, and that only extreme pH conditions could induce structural changes at the quaternary level.

### Simulated Gastrointestinal Digestion of PcPV2

**Figure 4:**
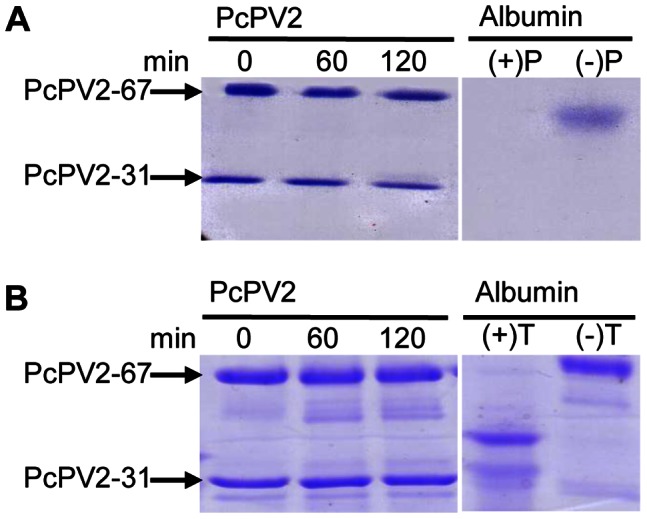
*In vitro* digestibility analyzed by SDS-PAGGE. A: Gastric digestion. Lanes 1–3, 0, 60 and 120 min of incubation, lanes 4 and 5 positive and negative control, respectively. B: Duodenal digestion. Lanes 1–3, 0, 30 and 120 min incubation; lanes 4 and 5, positive and negative controls, respectively. Positive control: Albumin with enzyme, negative control: albumin without enzyme.

In addition to extreme pH conditions, digestion by proteases is another stress that PcPV2 may endure to withstand the gastrointestinal tract of a predator. This hypothesis was tested both *in vitro* and *in vivo* (see below). By using a physiologically relevant digestion system, we found that PcPV2 was resistant to simulated gastric digestion for 2 h, as shown by SDS-PAGE (Figure 4A). After this simulated gastric digestion, the pH was adjusted to duodenal conditions, trypsin was added, and PcPV2 simulated intestinal digestion was performed for another 2 h. Again, PcPV2 showed no significant degradation (Figure 4B).

### Binding to the Intestinal Microvilli and Caco-2 Cells

**Figure 5:**
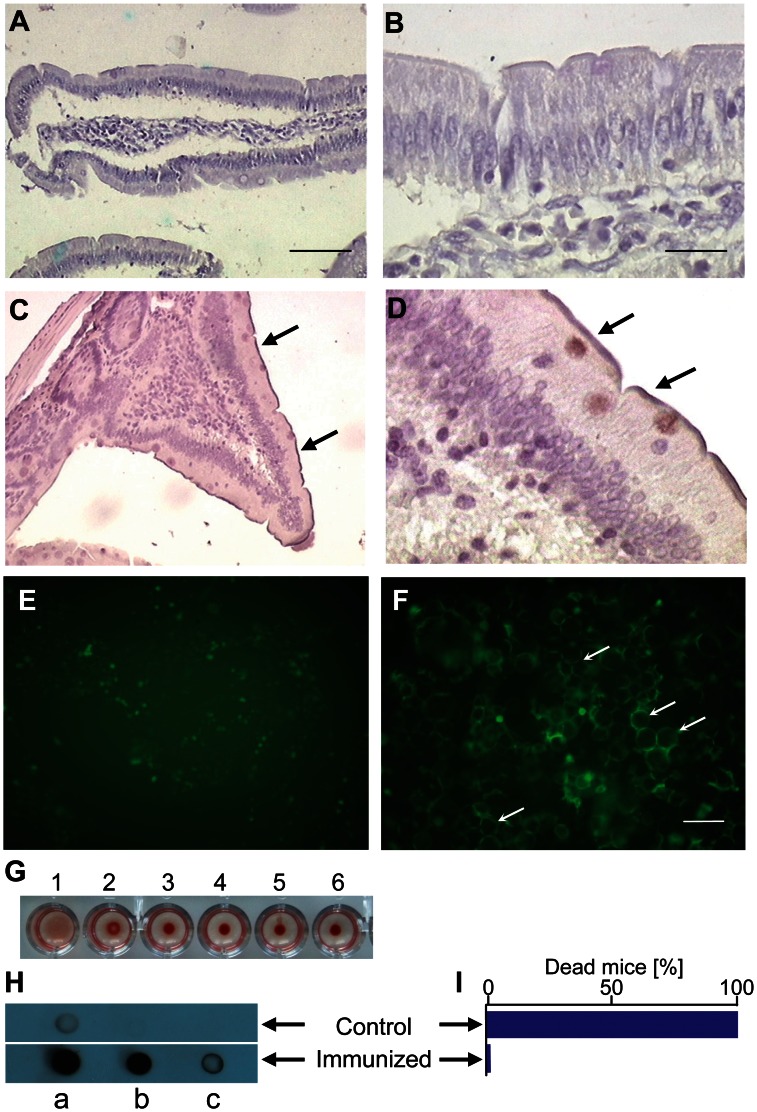
Binding to cells and presence in circulation of PcPV2. A,B,C,D: Immunolocalization of PcPV2 at the brush border of rat enterocytes. Rats were fed for 4 days on a diet without (A, B) or with (C, D) egg extracts containing the equivalent of 400 µg PcPV2. Arrows indicate anti-PcPV2 antibody binding to glycocalix. A, C: Bar 100 µm, B, D: Bar 15 µm. E, F: PcPV2 binding to intestinal cells in culture. Fluorescence microscopy of Caco-2 cells incubated for 1 h with Alexa-488 labeled BSA as control (E) or PcPV2 (F). Bar 25 µm. Arrows indicate specific toxin binding to cell surface. G: Hemagglutinating and hemolytic activity. Wells 1–5 two-fold serial dilution of 1.6 µg/ml PcPV2; 6 Control with buffer. H, I: Immunization of rodents by sublethal oral administration of PcPV2. Dot blot analysis of sera from rats orally immunized with PcPV2 (G). Strong positive immunoreactivity can be seen towards PcPV2 antigen. Dots a-c, sera from control or immunized rat diluted 1∶10, 1∶100, 1∶1000, respectively. PcPV2 was blotted onto nitrocellulose (1 µg in 5 µl PBS/spot). H. Effect of lethal i.p. dosis of PcPV2 on control and immunized mice survival. Graph I does not include error bars because all of the 6 immunized animals survived the intraperitoneal injection of a lethal concentration of the toxin, while all of the 6 control animals died after the injection.

Knowing that the PcPV2 toxin was able to withstand simulated gastrointestinal environmental conditions and that the light subunit has a putative carbohydrate binding domain (lectin-like structure), interactions between PcPV2 and the intestinal cell surface were screened using rat intestinal tissue and an enterocyte-like cell line. Immunlabeling with anti-PcPV2 antibodies showed positive reactions on the surface of villi in intestinal sections (Figure 5A,B,C,D), indicating that the toxin was specifically bound to mature enterocytes.

Protein localization studies using Caco-2 cells showed the presence of fluorescently labeled PcPV2 on the plasma membrane (Figure 5E,F). To rule out the possibility of nonspecific protein-cell interactions, Caco-2 cells were incubated with Alexa488-labeled BSA (Figure 5E), which showed a near absence of binding. Assuming that BSA binding represents the maximum nonspecific interaction between Caco-2 and proteins [35], this assay indicates that PcPV2 binding to cells by non-specific protein-cell interactions is extremely low.

### Hemagglutinating-hemolytic Activity of PcPV2

Taking into account the sequence homology of PcPV2-31 light subunit with tachylectins and the toxin binding capacity towards intestinal and Caco-2 cell membranes, we tested for hemagglutinating activity against rabbit erythrocytes. Positive reaction was observed above o.8 g/L of protein concentration or higher while no hemolytic activity could be detected up to 1.6 g/L of the toxin. (Figure 5G).

### Effect of Oral Immunization on Rats

For the neurotoxin to exert its effect, it is not only necessary that it reaches the intestine in a native conformation and that it interacts with epithelia but it also has to traverse the intestinal barrier and reach general circulation. To address this last issue, an indirect approach was employed evaluating the immune stimulating potential of ingested PcPV2 in Wistar rats that had been given oral sublethal doses of egg extracts as a source of PcPV2. Rats became immunized towards the toxin. Sera of immunized rats were positive in a dot blot performed using purified PcPV2 as antigen (Figure 5H). Furthermore, in another experiment, all mice subjected to an oral immunization protocol, survived an i.p injection of PcPV2 with a dose 8 times higher than its reported LD50, 96 h, without any altered clinical signs. On the other hand, all control animals died in less than 40 h, displaying the previously described symptomatology [17] (Figure 5I). These experiments using two different mammal species evidenced circulating antibody response to sublethal oral immunization with PcPV2.

## Discussion

### 1. PcPV2 Structure: A Novel Combination of MACPF and Tachylectin-like Neurotoxin

Several lectins have been reported in snail eggs, [36]. Lectin-like factors in *P. canaliculata* eggs were reported in the 1970s [37]. In the present work, we have identified a lectin-like domain in the egg neurotoxin PcPV2. The amino acid sequence deduced from full-length PcPV2 cDNA indicates that the PcPV2-31subunit belongs to a group of carbohydrate-binding proteins, the tachylectins, which are members of the F-type lectin family [36]. Tachylectins have been reported in the tissues of several invertebrates and some vertebrates [38], including a mollusk (*Mytilus galloprovinciallis)* [39]. In most cases, tachylectins seem to play a role in the innate immune system, including bacterial phagocytosis [40]. In addition, there are a few reports of tachylectins with bacteria-binding abilities in vertebrate [40] and invertebrate [41] eggs. Unlike these reported functions, the PcPV2 tachylectin-like domain might play a different role in apple snails (see below). This study provides the first molecular cloning of a tachylectin family member in gastropod mollusks.

The PcPV2-31 subunit is attached by a disulfide bond [18] to the PcPV2-67 subunit, which has high sequence identity with MACPF superfamily, one of the largest groups of pore forming molecules in both vertebrates and invertebrates [42], [43] and, like tachylectins, primarily involved in the immune response. In addition, some MACPF members play other roles such as neural migration, tumor suppression [34], or as a lethal toxin [44]. MACPF superfamily members have been described in abalone gastropods of the genus *Haliotis* [45], [46], thought to be involved in the immune system. In particular, PcPV2-67 amino acid sequence showed the greatest similarity to a pore-forming protein involved in the immune defense and development of another mollusk, the bivalve *M. galloprovincialis* [34] but, unlike the mussel MACPF it is assembled into a neurotoxic lectin-pore-forming heterodimer. To our knowledge this is the first study reporting a MACPF domain with neurotoxic activity [17]. Remarkably, PcPV2 does not share homology with other gastropod toxins namely the echotoxin-2, a pore-forming hemolysin from the triton *Cymatium parthenopeum* [47], the neurotoxin from the Crassispiridae *Crassispira cerithina* [48] or the neurotoxic conotoxins from the family Conidae [49].

A literature survey of lectins covalently attached to toxins showed that they are restricted to bacteria and plants, known as AB toxins [50]. In particular, there are very few AB toxins with a heterodimeric structure, namely plant type-2 RIPs (ribosome-inactivating proteins) and botulinum neurotoxins. Similar to PcPV2, these bacterial and plant toxins may enter the body through ingestion. These toxins have a carbohydrate-binding module (CBM) linked by a disulfide bridge to a toxic subunit as a heterodimer, and it is believed that the unifying feature of the CBMs in toxins is their function in delivering the toxic component of the protein to cell surfaces via a carbohydrate receptor–CBM interaction [51]. We explored whether this mechanism was possible in PcPV2 by analyzing if the toxin was structurally stable in a gastrointestinal environment and if it was able to bind to intestinal cells; both aspects are critical for toxicity (see next section).

### 2. PcPV2 Withstands Gastrointestinal Digestion, Binds to Intestinal Mucosa and would be able to Traverse the Intestinal Barrier

The PcPV2 neurotoxin displays structural stability in a pH range that falls within that of most vertebrate and invertebrate digestive tract fluids [52], [53]. Further, treatment of PcPV2 with gastrointestinal proteases, at biologically relevant concentrations revealed it is resistant, but not impervious, to pepsin and trypsin. In a physiological context, however, intestinal digestion would be even more limited since PcPV2 would be ingested together with large amounts of a trypsin inhibitor [13]. In addition, the capacity of PcPV2 to bind to intestinal cells is also indicative that it reaches the gut in an active conformation. The resistance gastrointestinal pH and digestive proteases are features that contribute to the defensive potential of many plant seed defensive proteins [13], like RIP dichain toxic lectins. [31], [54], [55].

**Figure 6:**
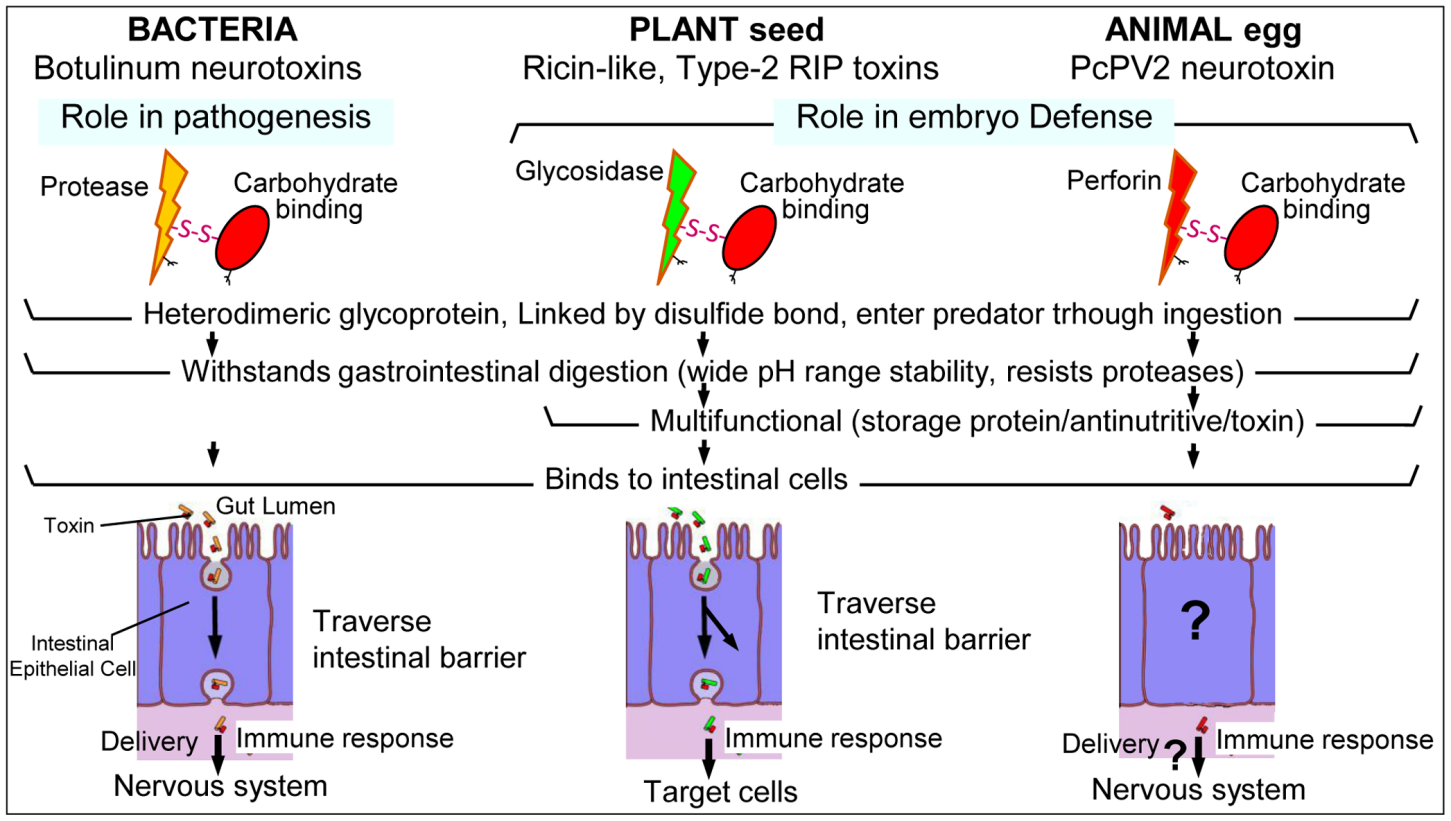
Similarities and differences in structure and function in dichain toxic lectins from bacteria, plants and apple snails. Question marks indicate unknown steps.

Toxins that enter the predator’s body by ingestion have first to bind to epithelial cells and then be transported into the general circulation. The neurotoxin showed hemaglutinating activity, indicating a functional lectin, together with a specific interaction with both duodenal epithelia and Caco-2 cells cell surfaces, suggesting the binding of the lectin moiety to glycoconjugates on enterocyte surfaces. The lack of hemolytic nor Caco2 or rat intestinal cell disruption activity would suggest that the MACPF domain is not active. On the basis of (1) the structural and functional similarities with plant and bacterial AB toxic lectins, (2) the thoroughly studied delivery role the lectin moiety always plays in plant and bacterial AB toxins and (3) the binding of PcPV2 to enterocytes without cell disruption, we can suggest that PcPV2 structure may be indicative of a delivery system for the MACPF toward the target cell surfaces. at the end of the journey, disrupting target neuronal membranes. (Figure 6 summarizes the similarities between these toxins). Future research will look at this matter.

Though we have no information on the mechanism by which PcPV2 would traverse the intestinal barrier, we observed that it was able to orally stimulate the immune system in rats and mice at concentrations much lower than the amount present in an egg clutch. The humoral response elicited resembles the reaction of mice and rats to numerous plant dietary lectins [56]–[58].

### 3. Ecological and Evolutionary Implications

Escaping predation is essential to survival for most animals and has resulted in the evolution of a great diversity of predator avoidance tactics. This report brings insights into the nature of apple snail egg defenses that suggest that the acquisition of this multifunctional storage protein may have conferred a survival advantage to the eggs, contributing to the virtual absence of predators.

The selection pressure exerted by predators and by the environment on apple snail eggs probably led to the acquisition of new features in their storage proteins. Data suggest they have co-opted into new functions, notably in embryo defenses against predation. In fact, the combination of two unrelated polypeptides resulted in a novel protein with neurotoxic properties, a feature not concurring with the roles classically ascribed to either animal lectins [36] or perforins [42]. Comparative analyses of the evolutionary origin of PcPV2 subunits indicate that both chains evolved separately, with an independent origin for MACPF-related proteins, and an early duplication for the tachylectin-like chain. Although there are reports of tachylectins and perforins in marine molluscs [39], [45] and this study reports tachylectin presence in Gastropoda, more comparative work is needed to identify when the unique combination appeared.

Apple snail egg defenses may be one of the few examples in animal chemical ecology highlighting the survival advantage of the acquisition of an antinutritive/neurotoxic defense which is an egg storage protein: By genetically encoding in the same complex these multifunctional proteins, synthesis is more cost-effective because females do not need to ingest toxic preys to endow eggs with chemical defenses. In this way *P. canaliculata* is able to circumvent allocation costs to defensive compounds, which may lower its relative fitness, by an efficient biochemical defense whose “leftovers” are nutritious proteins consumed by its embryos and hatchlings. This novel system might become useful to study the cost-benefit paradigm, central to most of functional biology [8], [13].

Finally, it is worth recalling that eggs and seeds are resting targets and, therefore, particularly vulnerable. In this regard, it is interesting to note that apple snail eggs and plant seed may have both developed (passive) biochemical defense systems to protect their embryos as an adaptation to predation, including the preferential accumulation of toxic lectins [54].

### Conclusions

This is, to our knowledge, the first evidence of a protein combining a tachylectin and MACPF subunits; two ancient and widely distributed proteins. Unlike their individual roles in the immune defense, joint together they resulted in a neurotoxin that might be involved in the biochemical defenses against predators.

This is also the first description in animals of a defense system employed by plants against predators. It suggests unforeseen similarities between poisonous seeds and poisonous eggs, indicating that protection mechanisms thought to be confined to plants are also part of an animal’s defensive repertoire.

This study also provides evidence of the first steps of the neurotoxin’s journey towards its target, indicating that it is capable of withstanding the gastrointestinal tract, interact with epithelial cells and reach circulation.

The discovery brings insights into the nature of apple snail egg defenses that suggest that the acquisition of this multifunctional storage protein may have conferred a survival advantage to the eggs, contributing to the virtual absence of predators, setting the stage for further investigations of the evolution of defensive strategies against predation.

## Supporting Information

Figure S1 **2-DE analysis of native (A) and dissociated (B) PcPV2.**
(TIF)

Figure S2 **Alignment between PcPV2-67 and the sequences of the five more related MACPF members from** **Figure 2** **.** Light red box indicates MACPF signature; black box, MACPF domain.
(TIF)

Figure S3 **Pair Distance Distribution Function (PDDF) of PcPV2 obtained from SAXS data at different pH values.** The PDDF is the probability of finding a given point-to-point distance within the boundaries of the molecule. The vertical lines indicate the mean Paired Distance obtained from the graphic.
(TIF)
