# Supplementary figures and images for: Novel Animal Defenses against Predation: A Snail Egg Neurotoxin Combining Lectin and Pore-Forming Chains That Resembles Plant Defense and Bacteria Attack Toxins
Source: PLoS One. 2013 May 30;8(5):e63782. doi: 10.1371/journal.pone.0063782 (PMC3667788; doi:10.1371/journal.pone.0063782)

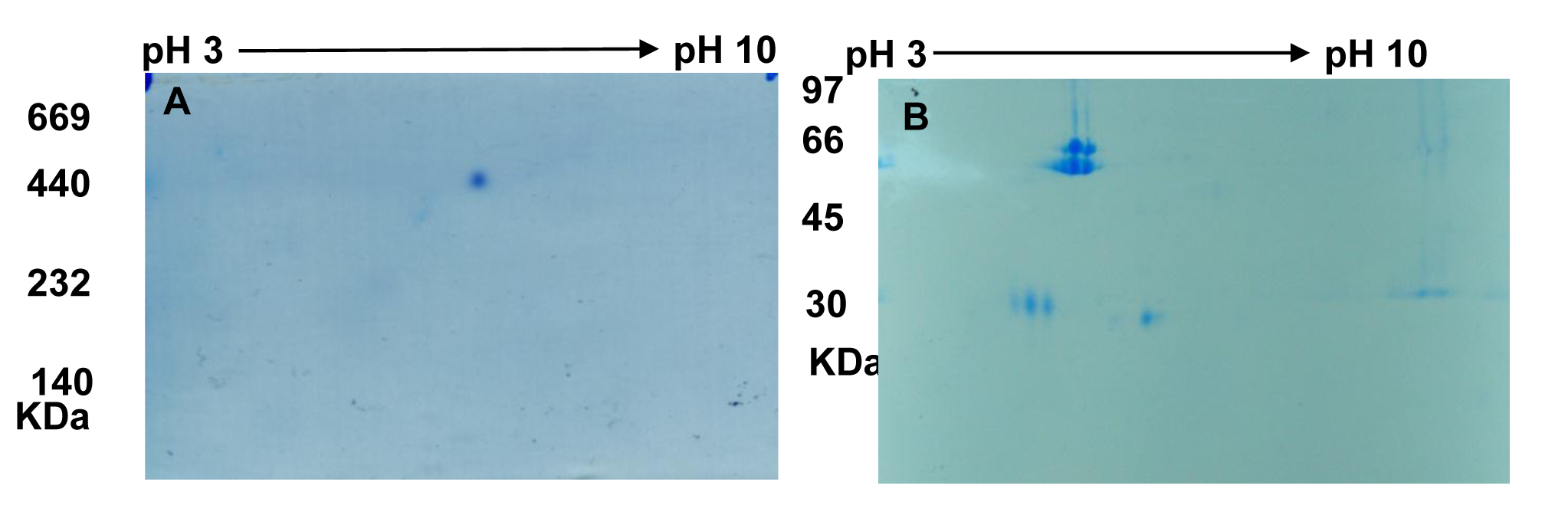

Supplement: Figure S1 — 2-DE analysis of native (A) and dissociated (B) PcPV2. (TIF) [file pone.0063782.s001.tif]

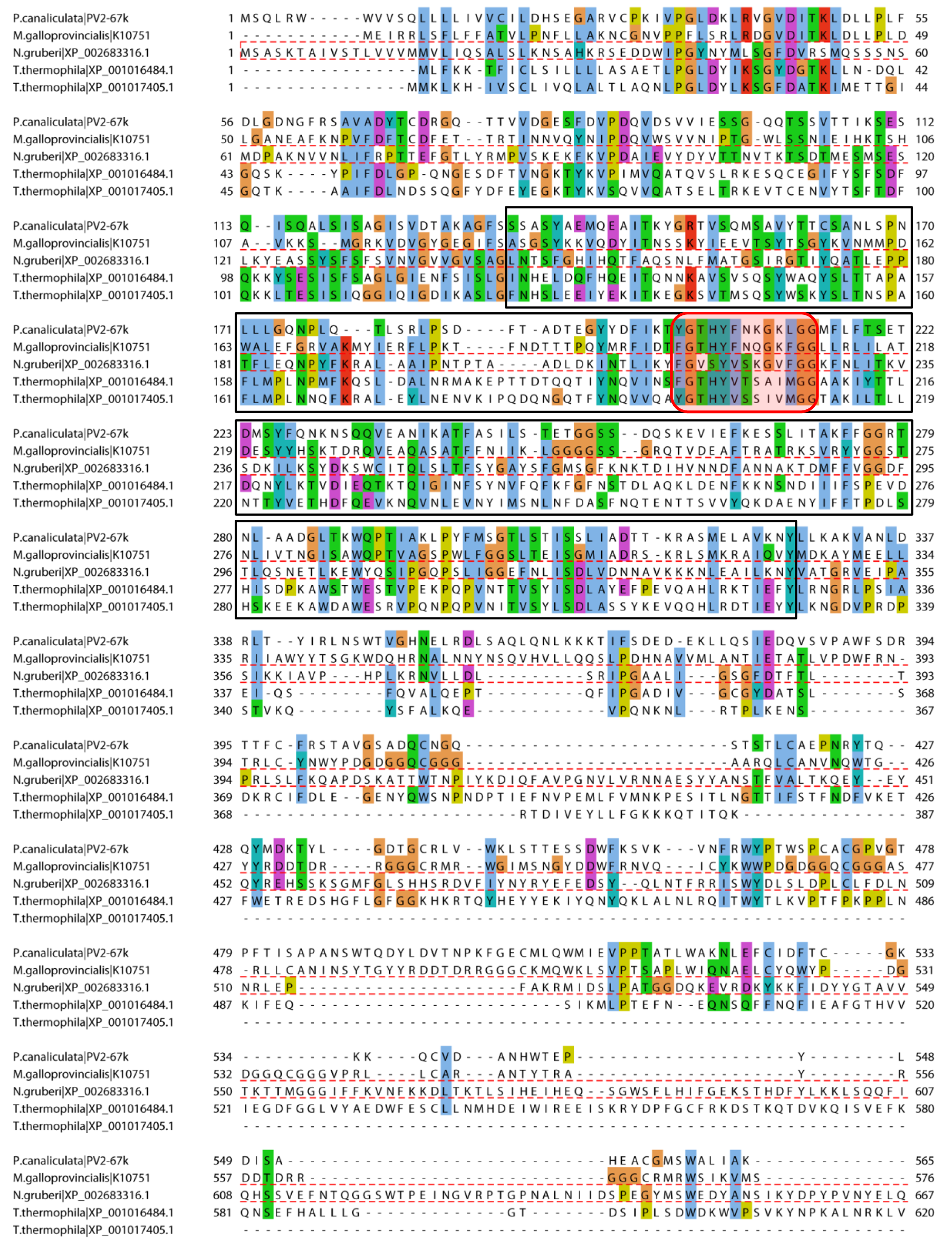

Supplement: Figure S2 — Alignment between PcPV2-67 and the sequences of the five more related MACPF members from Figure 2 . Light red box indicates MACPF signature; black box, MACPF domain. (TIF) [file pone.0063782.s002.tif]

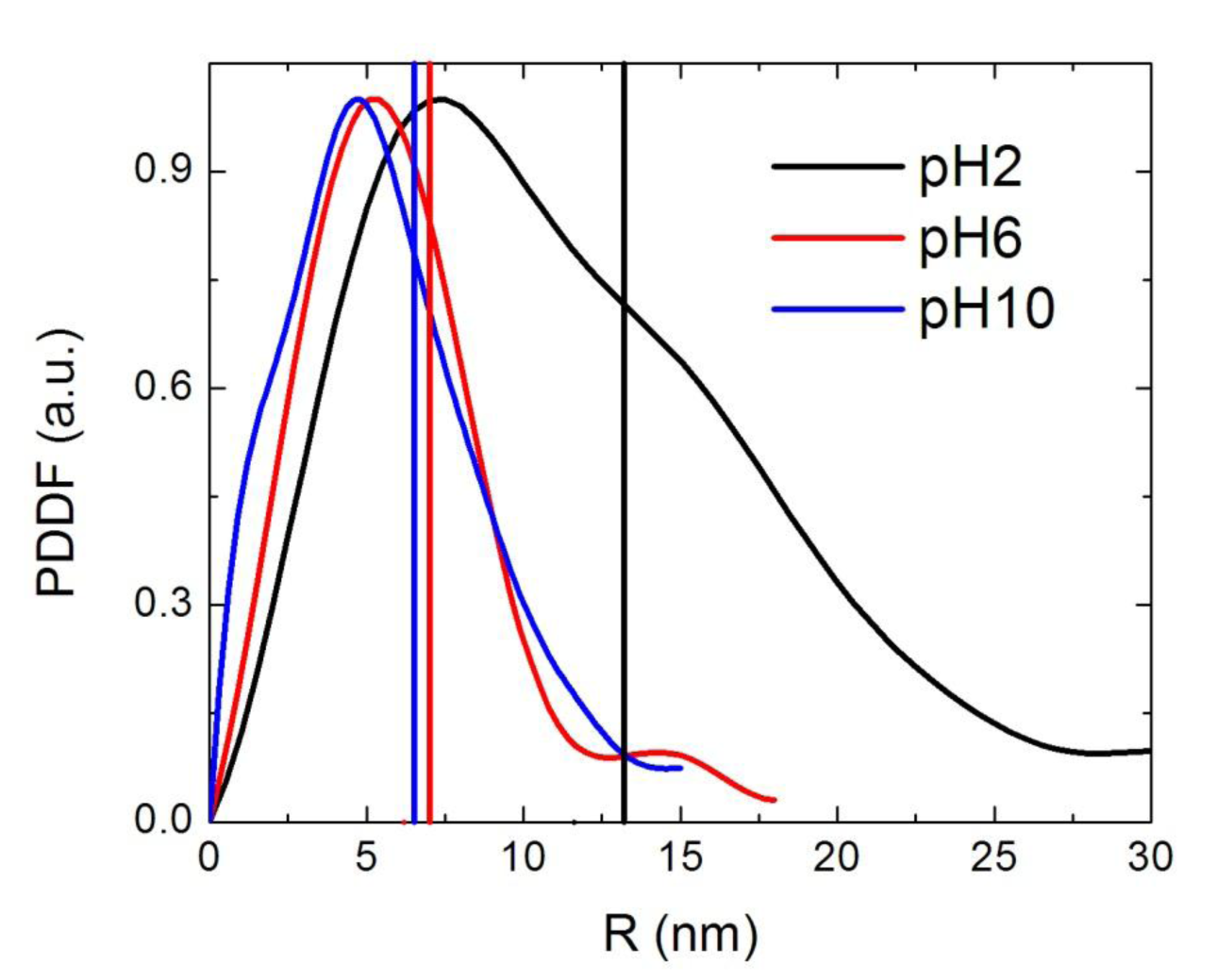

Supplement: Figure S3 — Pair Distance Distribution Function (PDDF) of PcPV2 obtained from SAXS data at different pH values. The PDDF is the probability of finding a given point-to-point distance within the boundaries of the molecule. The vertical lines indicate the mean Paired Distance obtained from the graphic. (TIF) [file pone.0063782.s003.tif]
